# Directing Trophic Divergence in Plant-Pathogen Interactions: Antagonistic Phytohormones With NO Doubt?

**DOI:** 10.3389/fpls.2020.600063

**Published:** 2020-12-03

**Authors:** Shuanglong Huang, Xuehua Zhang, W. G. Dilantha Fernando

**Affiliations:** Department of Plant Science, University of Manitoba, Winnipeg, MB, Canada

**Keywords:** trophic divergence, hormones, nitric oxide, plant-pathogen interactions, biotrophs, necrotrophs, hemibiotrophs

## Abstract

A fundamental process culminating in the mechanisms of plant-pathogen interactions is the regulation of trophic divergence into biotrophic, hemibiotrophic, and necrotrophic interactions. Plant hormones, of almost all types, play significant roles in this regulatory apparatus. In plant-pathogen interactions, two classical mechanisms underlying hormone-dependent trophic divergence are long recognized. While salicylic acid dominates in the execution of host defense response against biotrophic and early-stage hemibiotrophic pathogens, jasmonic acid, and ethylene are key players facilitating host defense response against necrotrophic and later-stage hemibiotrophic pathogens. Evidence increasingly suggests that trophic divergence appears to be modulated by more complex signaling networks. Acting antagonistically or agonistically, other hormones such as auxins, cytokinins, abscisic acid, gibberellins, brassinosteroids, and strigolactones, as well as nitric oxide, are emerging candidates in the regulation of trophic divergence. In this review, the latest advances in the dynamic regulation of trophic divergence are summarized, emphasizing common and contrasting hormonal and nitric oxide signaling strategies deployed in plant-pathogen interactions.

## Introduction

Plant pathogens are often clustered into three types: biotrophs, hemibiotrophs, and necrotrophs, based on their lifestyles, notably the strategies of nutritional acquisition and structural changes (Perfect and Green, 2001; Kraepiel and Barny, 2016). Biotrophs establish trophically in the apoplast and assimilate nutrients directly from the living host tissues without inducing programmed cell death (PCD), or asymptomatically. On the contrary, necrotrophs break plasma membranes and execute PCD in the host prior to nutrient uptake, or destructively. Hemibiotrophs, traditionally believed to share the trophic features of both biotrophs and necrotrophs, emulate the characteristics of biotrophic pathogens in the first phase and those of necrotrophs in the second phase. The morphological landmark of the trophic switch during the infection process is the growth of thick primary hyphae in the biotrophic phase followed by the formation of thin secondary hyphae in the necrotrophic phase (Chowdhury et al., 2017). To complete their lifecycles successfully, plant pathogens also evolve to selectively activate genes either for adapting to colonize and disintegrate the intercellular matrix or for breaching cell walls and cellular compartments in biotrophic and necrotrophic interactions, respectively (Koh and Somerville, 2006; Di Pietro et al., 2009; Meinhardt et al., 2014; Fernandes et al., 2017). The underlying mechanisms for the trophic switch, or the transition from biotrophic to necrotrophic phase during plant hemibiotrophic interactions remain largely unknown, though their execution requires the programming and reprogramming of specific secreted effector proteins (Kelley et al., 2010; Lee and Rose, 2010; Vargas et al., 2012; Yang et al., 2013).

Hormones, the multifaceted signal molecules controlling plant growth and development, are also essential regulators of pathogen-triggered programmed cell death (pPCD), or plant immunity-associated cell death (Spoel and Dong, 2008; Huysmans et al., 2017). Strikingly, the machinery underlying hormone regulated pPCD in the host during plant-pathogen interactions is manifested by the infection strategy of the pathogen, or more specifically, either biotrophic or necrotrophic (Glazebrook, 2005). Earlier studies on hormonal regulation in trophic divergence have illustrated that salicylic acid (SA), jasmonic acid (JA), and ethylene (ET) play essential roles in navigating biotrophic and necrotrophic interactions (Glazebrook, 2005; Tsuda et al., 2009, 2013). NPR1, the master regulator of SA signaling, also mediates systemic acquired resistance (SAR), the induced immune response offers protection counteracting broad-spectrum pathogens (Fu and Dong, 2013). However, SAR seems to be more effective against biotrophs and hemibiotrophs than necrotrophs (Hammerschmidt, 2009; Luna et al., 2012). More recently, progress has been made in the understanding of hormonal regulation of trophic divergence via integrated approaches (Robert-Seilaniantz et al., 2011a; Denancé et al., 2013; López-Ráez et al., 2017). The focus has seemingly expanded toward the involvement of other hormones, such as auxins, cytokinins (CKs), abscisic acid (ABA), gibberellins (GAs), brassinosteroids (BRs) and strigolactones (SLs), and signaling molecules such as nitric oxide (NO). Here, we briefly revisit the SA and JA/ET antagonism that is well-established (Robert-Seilaniantz et al., 2011a), and intensively anatomize these emerging players in directing trophic divergence and infer future directions for this area.

## Centrality of Salicylic Acid and Jasmonic Acid/Ethylene Antagonism in Trophic Divergence

The regulation of plant defense is directed by complex signaling pathways that are often interconnected, among which, SA and JA/ET are the most well-studied antagonistic hormone pairs in host-pathogen interactions. These SA- and JA/ET-dependent pathways, and their crosstalk defense mechanisms operate acutely in response to a single pathogen or multiple pathogens of different trophic phases and types (Kloek et al., 2001; Spoel et al., 2003, 2007; Zhao et al., 2003). Other than the specifically inducible defense of SA and JA/ET against most biotrophic and necrotrophic pathogens, the concerted action of both SA and JA/ET was well shown in hemibiotrophic interactions in *Arabidopsis thaliana*-*Pseudomonas syringae* (Spoel et al., 2003), *A. thaliana-Fusarium oxysporum* (Anderson et al., 2004; Kazan and Manners, 2008; Grant et al., 2013; Kazan and Lyons, 2014) and *Brassica napus*-*Leptosphaeria maculans* (Becker et al., 2017; Zou et al., 2020) pathosystems. The SA receptor NPR1 (Wu et al., 2012), and potentially NPR2 (Castelló et al., 2018), is the key to this crosstalk, modulating the SA-mediated activation of PR genes such as the conserved PR1, but also the suppression of JA biosynthetic and responsive genes like PDF1.2 in *A. thaliana* against *P. syringae* (Spoel et al., 2003) and *F. oxysporum* (Anderson et al., 2004). Interestingly, these JA-responsive pathogen defense genes are negatively regulated by the basic helix-loop-helix Leu zipper transcription factor MYC2/JIN1 in the *A. thaliana-F. oxysporum* pathosystem (Anderson et al., 2004; Lorenzo et al., 2004; Dombrecht et al., 2007). Maneuvered in a tissue specific fashion, such antagonism between SA and JA/ET, is also applicable in the defense commutation between biotrophic and necrotrophic pathogens (Spoel et al., 2007). The induction of SA by the virulent strain of hemibiotrophic *P. syringae* in the host suppresses JA signaling pathways, manifesting the elevated susceptibility to the necrotrophic pathogen *Alternaria brassicicola* in local infection tissues but not systematic tissues (Spoel et al., 2007). However, it is important to keep in mind that the perceptible effect of SA and JA/ET on trophic divergence does not exclude the crosstalk between SA and JA/ET (Li et al., 2019), and most likely it is a collective outcome orchestrated by other hormones that will be discussed in the following sections.

## Additional Antagonistic Hormones and Trophic Divergence

Hormone crosstalk is one of the major strategies that plants utilize in prioritizing growth or defense (Huot et al., 2014), and such balancing is reminiscent of trophic divergence. In addition to the conventional defense hormones SA and JA/ET regulating the variance of biotrophic and necrotrophic interactions, auxin and cytokinin, ABA, and GA are emerging as newer antagonistic players. To highlight their implications, our focus in this section is pointing out the involvement of these antagonistic hormones, as well as their interactions with SA and JA/ET in navigating trophic divergence.

### Auxins and Cytokinins in Trophic Divergence

While auxins and CKs are antagonistic in the homeostasis of cell division and differentiation in root meristem niche (Su et al., 2011), it remains largely unknown, whether such antagonism is also present and how they may interact with SA and JA/ET pathways in trophic divergence in plant-pathogen interactions. Independent research has demonstrated that auxin negatively correlates with ETI (effector-triggered immunity) and PTI (pattern-triggered immunity) mediated susceptibility via manipulations in auxin sensitivity or auxin responsive genes (Chen et al., 2007; Kazan and Lyons, 2014). Interestingly, in the *P. syringae*-*A. thaliana* hemibiotrophic interaction, attenuation of PIN1-mediated auxin transport was also associated with the increase in host susceptibility (Nomura et al., 2006; Tanaka et al., 2009; Kazan and Lyons, 2014). In the same hemibiotrophic pathosystem, overexpression of GH3.5, the bifunctional modulator of both auxin and SA (Zhang et al., 2007), has enhanced host resistance likely by suppressing auxin biosynthesis while promoting SA response. Conversely, elevating auxin biosynthesis via overexpression of the auxin (indole-3-acetic acid, IAA) biosynthesis gene YUCCA1 (YUC1) was able to escalate host susceptibility independent of SA response in Arabidopsis plants infested with the hemibiotrophic *P. syringae* inoculum (Mutka et al., 2013). Moreover, further studies indicated that overexpression of the auxin receptor AFB reduces host susceptibility in a biotrophic interaction excited by *Hyaloperonospora arabidopsidis* but exerts no effect on host susceptibility in a necrotrophic interaction by *A. brassicicola* (Robert-Seilaniantz et al., 2011b). These studies have suggested that trophic divergence could be affected by auxin biosynthesis, signaling, and transport. It is worth noting that the navigation of biotrophic interactions by auxin could be dependent or independent of SA. On the other hand, auxin may navigate the necrotrophic interactions, as illustrated in the *A. thaliana-A. brassicicola* pathosystem, promoting auxin biosynthesis and repressing auxin transport, synergistically with the JA/ET pathways via inducing the expression of PDF1.2 and HEL (Qi et al., 2012), which requires more detailed investigations.

Similar to auxins, CKs are also involved in the navigation of trophic divergence, but the effect on host defense response is diversified. Independent studies investigating hemibiotrophic interactions in the *A. thaliana-P. syringae* and *Oryza sativa-Magnaporthe oryzae* pathosystems indicate that CK acts synergistically with SA and results in aggrandized host resistance when CK is increased (Naseem et al., 2012; Jiang et al., 2013), possibly dependent of the SA receptor NPR1 (Choi et al., 2011). In biotrophic interactions rendered by *H. arabidopsidis*, a negative feedback of SA on CK is instead established in the regulation (Argueso et al., 2012; Naseem et al., 2014). In this specific interaction, higher levels of CK reduced host susceptibility while lower CK levels favored host susceptibility (Argueso et al., 2012). Similarly, in necrotrophic interactions, the effect of CK on the defense response also operates in concentration-dependent fashion. Elevated expression of CK response regulators ARR and IPT genes increases CK levels and enhances host resistance (Choi et al., 2011), while overexpression of CKX4 lowers CK levels and shows a reverse phenotype against the same necrotrophic pathogen (Choi et al., 2010).

### Abscisic Acid and Gibberellins in Trophic Divergence

The third antagonistic hormone pair ABA and GA, eminent in directing seed dormancy and germination (Liu and Hou, 2018), also engages in trophic divergence. As compared to their explicit roles in regulating plant growth and development and abiotic stress response (Shu et al., 2016), the involvement of ABA and GA in navigating trophic divergence is less explored, but mounting evidence has revealed some regulatory patterns in both dicots and monocots challenged by biotrophic, necrotrophic, and hemibiotrophic interactions.

By interacting with SA and JA/ET, ABA is versatilely involved in the regulation of host defense response in biotrophic, necrotrophic, and hemibiotrophic interactions (Denancé et al., 2013). Intriguingly, while directing the trophic interactions, ABA appears to negatively regulate the defense response in biotrophic interactions delineated by *Triticum aestivum-F. graminearum* (Buhrow et al., 2016) and *A. thaliana-Golovinomyces cichoracearum* (Xiao et al., 2017) pathosystems. And, hemibiotrophic interactions in the *A. thaliana-P. syringae* pathosystem (Mohr and Cahill, 2003) with ABA biosynthesis and signaling impairing shown to elevate host resistance. It was speculated that such navigation was executed antagonistically with SA (Denancé et al., 2013; Han and Kahmann, 2019). Dissimilarly, the role of ABA in directing necrotrophic interactions is dichotomous. Earlier studies revealed that ABA may negatively modulate necrotrophic interactions antagonizing with JA/ET signaling, as well exemplified in Arabidopsis plants confronted by *Plectosphaerella cucumerina* (Sánchez-Vallet et al., 2012) and *F. oxysporum* (Anderson et al., 2004). In the latter, the antagonistic effects between ABA and JA/ET employed in the host was further explored to negatively regulate disease resistance against *F. oxysporum* modulated by MYC2 (Anderson et al., 2004). Surprisingly, ABA also negatively regulate host resistance suppressing SA in the necrotrophic interaction mediated by the *Lycopersicon esculentum-Botrytis cinerea* pathosystem (Audenaert et al., 2002). In contrast, ABA was also shown to positively regulate resistance in Arabidopsis plants challenged by necrotrophic pathogens *Pythium irregulare* (Adie et al., 2007), *A. brassicicola* (Flors et al., 2008; Fan et al., 2009), *B. cinerea* (Garcia-Andrade et al., 2011), and *P. cucumerina* (Hernandez-Blanco et al., 2007; Garcia-Andrade et al., 2011), possibly mediated by the ET signaling pathway (De Vleesschauwer et al., 2010).

Similar to its multifaceted functions in plant growth and development, GA is also entailed on trophic divergence. Independent studies have implied that GA acts in a dicot- and monocot-specific manner and contributes to maneuvering trophic divergence (De Bruyne et al., 2014). In dicots, at least in *A. thaliana*, loss-of-function in DELLAs and exogenous applications of GA were shown to enhance host resistance to hemibiotrophic *P. syringae* attacks but impair host resistance counteracting against the necrotrophic pathogen *A. brassicola* via a DELLA-dependent pathway (Robert-Seilaniantz et al., 2007; Navarro et al., 2008). In several monocot systems inclusive of wheat, barley, and rice, however, independent observations suggest that GA appears to play a dichotomous role in the regulation of trophic divergence. Specifically, wheat and barley plants with gain-of-function in DELLAs elevated host susceptibility to biotrophic *Blumeria graminis* while promoting host resistance against necrotrophic *Oculimacula acuformis* and *O. yallundae* and hemibiotrophic *F. graminearum* (Saville et al., 2012). Opposite to the findings in wheat and barley, enriched studies in rice demonstrate that the DELLA protein SLR1 indulges host resistance against biotrophic and hemibiotrophic pathogens (Yang et al., 2012; Qin et al., 2013; De Vleesschauwer et al., 2016), but suppresses host susceptibility to necrotrophic pathogens (De Vleesschauwer et al., 2012). These studies clearly demonstrated that GA operates irreconcilably in the trophic divergence between dicots and monocots, and the contradiction among them, especially its role in wheat and barley discrepantly to rice, is likely the interactive outcome of GA specifically with SA or JA/ET (Navarro et al., 2008; De Vleesschauwer et al., 2016). It is also noteworthy that such a unique mechanism in trophic divergence is regulated dependently of signaling pathways mediated by DELLAs, negative regulators of GA signaling (De Bruyne et al., 2014; De Vleesschauwer et al., 2016).

## Brassinosteroids and Trophic Divergence

Substantial progress has been made in the roles of BRs in plant growth and development and plant response to abiotic stresses and pathogen attacks. Molecular mechanisms illustrating multidirectional BR signaling were well-characterized (Nolan et al., 2020), while the involvement of BRs in plant-pathogen interactions is relatively ambiguous, as contradicting observations were obtained from earlier studies (De Bruyne et al., 2014). With regard to trophic divergence, the regulation by BRs becomes more intricate. In biotrophic relationships, a possible pattern could be perceived from most studies showing that elevated levels of BRs in the host seems to enhance resistance against biotrophic pathogens including *Oidium neolycopersici* (Nakashita et al., 2003). Contrarily, it is difficult to form a consensus pertaining to the roles of BRs in hemibiotrophic and necrotrophic interactions. Independent studies demonstrated that higher contents of BRs could increase either resistance against hemibiotrophic *F. culmorum* (Albrecht et al., 2012; Ali et al., 2013) or susceptibility against necrotrophic *P. graminicola* through crosstalk with SA and GA (De Vleesschauwer et al., 2012). However, disruption in BR receptor BRI1 has been shown to promote host (*Brachypodium distachyon* and *Hordeum vulgare*) resistance against hemibiotrophic *M. oryzae* (Goddard et al., 2014). Intriguingly, the possible mimicking effect between BR and GA (De Bruyne et al., 2014) allows us to speculate on their crosstalk in trophic divergence likely mediated by the BR signaling transcription factor BZR1 (Lozano-Durán et al., 2013) and DELLA protein SLR1 (De Vleesschauwer et al., 2012). It is foreseeable from these studies that BRs play important roles in trophic divergence. However, questions such as whether BR-mediated regulation of trophic divergence is pathosystem-specific, and whether the observed contrasting phenotypes are an outcome of either the spatiotemporal distributions of BRs or the crosstalk of BRs with other hormones such as auxins given their shared roles in cell expansion and proliferation (Hardtke et al., 2007), remain largely unanswered.

## Strigolactones and Trophic Divergence

In addition to the above-mentioned hormones, SLs are important regulators of plant growth and development (Waters et al., 2017), while limited information is available related to their roles in plant defense against pathogens (Pandey et al., 2016). Forward genetics approaches have shown that strigolactone deficiency has a contrasting effect on host susceptibility in pathogens of different lifestyles. For instance, when challenged by necrotrophic *B. cinerea*, tomato Slccd8 mutants underwent more severe disease development and disease symptoms (Torres-Vera et al., 2014). Likewise, host plants with knockout of CCD7 and CCD8 become more vulnerable to the necrotrophic pathogen infection during *Orobanche ramosa*-*Sclerotinia sclerotiorum* interactions (Decker et al., 2017) using GR24, one of the SL analogs (Zwanenburg and Pospísil, 2013). Thus, it is reasonable to hypothesize a possible link of GR24 to trophic divergence, especially in necrotrophic interactions, though high variability of *in vitro* morphogenesis of some necrotrophic pathogens (López-Ráez et al., 2017). When challenged by hemibiotrophic *F. oxysporum*, pea plants with different SL levels did not exhibit any difference in disease development or disease symptoms (Foo et al., 2016). Strikingly, independent studies on the hemibiotrophic *A. thaliana*-*P. syringae* pathosystem showed that, mutations in SL biosynthesis (MAX3 and MAX4) and perception (MAX2) manifest host sensitivity to the disease, likely via a signaling pathway independent of ABA signaling pathways (Piisilä et al., 2015; Kalliola et al., 2019). On the other hand, Arabidopsis plants defective in SL biosynthesis and signaling become more susceptible when challenged by biotrophic *Rhodococcus fascians*, with the possibility of crosstalk with CKs involving receptors AHK3 and AHK4 (Stes et al., 2015). This evidence clearly shows that SLs are involved in trophic divergence, and their roles might be pathosystem-specific. This is possibly the outcome of interactions of the lifestyle of the pathogen, the levels of SLs in the host dependent or independent of other hormones like ABA, CK, GA, JA and SA (Omoarelojie et al., 2019), and the environmental conditions, meaning that more studies are required to determine their roles in trophic divergence.

## Emerging Roles of Nitric Oxide in Trophic Divergence

It is well-known that nitric oxide is a multitasked signaling molecule in plant biology from development (Huang et al., 2014) to defense (Domingos et al., 2015). During plant-pathogen interactions, cellular levels of NO are believed to facilitate early establishment of the pathogen but also restrict further pathogenic infections (Martínez-Medina et al., 2019). The versatility of NO in trophic divergence is even more complex, but studies detailing in pathogens with different trophic lifestyles have shed light on some of the common and differential patterns.

Despite their explicit differences in trophic characteristics, biotrophs, hemibiotrophs, and necrotrophs are able to produce NO. The generation of NO could occur distinctly in several vegetative tissues, including mycelia in biotrophic *Blumeria graminis* and *O. neolycopersici* and hemibiotrophic *M. oryzae* (Prats et al., 2008; Piterková et al., 2009; Samalova et al., 2013), hyphae in biotrophic *Bremia lactucae* (Sedlářová et al., 2011), and spores in necrotrophic *B. cinerea* (Floryszak-Wieczorek et al., 2007). Similar to ROS (Heller and Tudzynski, 2011), these studies suggest that NO also plays an important part during fungal morphogenesis and reproduction (Cánovas et al., 2016), which requires further investigations to reveal its specific roles contributing to trophic classification.

Strikingly, spatiotemporal distributions of pathogen-triggered NO and their concentrations appear to participate in the direction of trophic divergence. At the outset of biotrophic contact with the host, NO is induced and present in both compatible and incompatible interactions (Sedlářová et al., 2011), while it is only detectable in the compatible interactions during the necrotrophic contact (van Baarlen et al., 2004; Turrion-Gomez and Benito, 2011). Interestingly, a relative higher concentration of the induced NO, likely via the reduction of S-nitrosoglutathione (Zhang et al., 2015), is commonly observed in the compatible interactions elicited by the biotrophic pathogen *B. lactucae* (Sedlářová et al., 2011) and the necrotrophic *B. cinerea* (Turrion-Gomez and Benito, 2011). In the incompatible interactions, a lower concentration of the incited NO was often found to encompass the infection site where hypersensitive cell death occurs to prevent an outward disease spread (Piterková et al., 2011; Sedlářová et al., 2011). However, with limited literature available, these independent studies should be interpreted cautiously, as the possible role of NO in trophic divergence might be pathosystem-specific. In addition, possible crosstalk of NO with other defense components such as ROS (Bellin et al., 2013) and hormones including but not limited to the SA and JA/ET antagonism (Bari and Jonathan, 2009; Mur et al., 2013) might be able to mechanistically explain trophic divergence.

## Conclusion and Perspective

**FIGURE 1:**
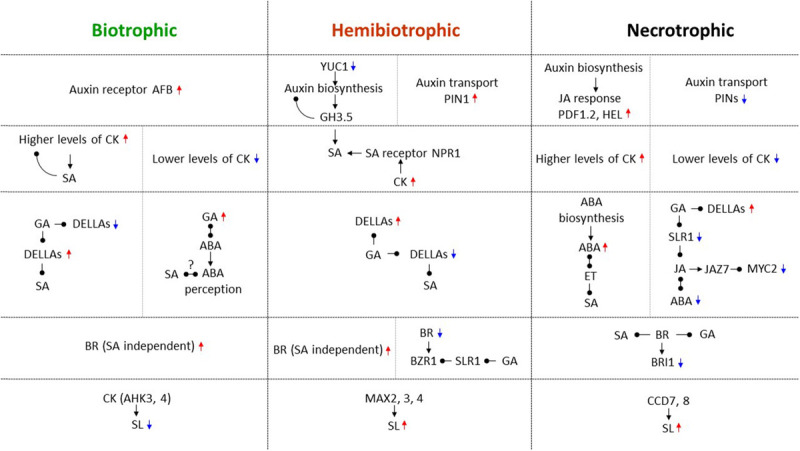
Hormone crosstalk in directing biotrophic, hemibiotrophic, and necrotrophic interactions. During trophic divergence, SA orchestrates both biotrophic and hemibiotrophic interactions, while JA/ET orchestrates both necrotrophic and hemibiotrophic interactions. By interacting with SA, JA/ET, and other hormones, auxin, CK, ABA, GA, BR, and SL as well as their important biosynthesis, transport and response genes, receptors, regulatory components, and transcription factors are all involved in directing trophic divergence, and their crosstalk are widely diversified across these three trophic interactions. Red upward triangle arrows are used to indicate positive regulators for host immunity, and blue downward triangle arrows for negative regulators for host immunity in each specific trophic interaction. Black triangle arrows are denoted for positive regulations in the pathway, while black round arrows for negative regulations in the pathway. Question marks designate potential regulations that require further investigations. Trophic divergence by different hormones are separated by horizontal dotted lines, while different regulatory modules within a trophic interaction are separated by vertical dotted lines.

From the studies described above, it is apparent that complex signaling pathways mediated by hormones and NO regulate trophic divergence. Identified as the first “defense” hormone antagonism, SA and JA/ET are a central part of navigating biotrophic and necrotrophic interactions. Two additional antagonistic hormone pairs, auxins and CKs, as well as ABA and GA, classical regulators of plant growth and development, have also played important roles in the regulation of trophic divergence. Moreover, two relatively new hormones, brassinosteroids and strigolactones contribute substantially to directing trophic divergence. The versatile signaling molecule NO has emerged as a key component in the direction of trophic divergence. Similar to plant growth and development, hormonal crosstalk and interplays between hormones and NO (Sanz et al., 2015) are also required for directing plant-pathogen interactions, especially trophic divergence. In the above sections, we anatomize these “newer” multifaceted players by highlighting their specific regulatory roles, and more importantly, accentuating their crosstalk with SA and JA/ET and/or with each other in trophic divergence. It is apparent that these hormones often operate in conjunction with each other and fine tune trophic divergence. The choice of which hormones to be involved, their concentrations (either high or low), and their interaction modes (either antagonistic or synergistic) are important for trophic divergence. The roles of hormone crosstalk are so diversified in navigating trophic divergence and details are summarized in Figure 1. One of the best examples is that JA, auxin, ABA, GA, and BR were shown to interact with the DELLA protein SLR1 in response to biotic and abiotic stresses (De Bruyne et al., 2014), and it may also be the case in the regulation of trophic divergence. Another notion to be ingrained in the understanding of trophic divergence is, while hormones largely regulate trophic divergence in concerted actions, the levels of hormones such as auxin, CK, and SA are for example changed due to gall formation, in the biotrophic *B. napus*-*Plasmodiophora brassicae* interaction (Prerostova et al., 2018), and such a unique reciprocal regulation module might be ubiquitous in hormone modulation of trophic divergence.

Collectively, these studies have suggested that by interacting antagonistically or synergistically, hormones and NO may act as important regulators in trophic divergence. The pathosystem itself, the concentration and spatiotemporal distribution of the involved hormones and NO, and the timing of their actions are among those key modulators that determine the navigation of trophic divergence. Hormones and NO may also coordinate with ROS (Yoda et al., 2003; Torres et al., 2006; Shetty et al., 2007; Lyons et al., 2015) and maneuver trophic divergence. Due to the complex interactions involved, global gene expression analysis at transcriptional, translational, or post-translational levels may broaden our understanding of how hormones and NO regulatory networks specify trophic divergence. Meanwhile, when a given component is amended genetically or pharmaceutically during plant-pathogen interactions, especially during hemibiotrohic interactions that endure a trophic switch accompanied by the reprogramming of gene regulatory networks, detailed time-course studies may be necessary to dissect its exact role in trophic divergence. To this end, it would be interesting to investigate more host systems challenged by biotrophic and necrotrophic pathogens, as this would allow us to have a comprehensive model of hormone and NO signaling in trophic divergence.

## Author Contributions

All authors listed have made a substantial, direct, and intellectual contribution to the work, and approved it for publication.

## Conflict of Interest

The authors declare that the research was conducted in the absence of any commercial or financial relationships that could be construed as a potential conflict of interest.
